# Items

**Published:** 1903-02

**Authors:** 


### ITEMS.

The Lambert Pharmacal Company, of St. Louis, has sent us a very handsome calendar stand, mounted in strong leather, and equipped with calendar cards for the year. We offer our thanks for the courtesy.

The Enno Sander Prize, of the Association of Military Surgeons of the United States for 1903, will be awarded to the author of the best essay on The differential diagnosis of typhoid fever in its earliest stages.

The board of award will consist of Dr. Austin Flint, of New York; Colonel Calvin DeWitt of the Army; Prof. Victor C. Vaughan, of Ann Arbor. Full information concerning the contest may be obtained from Major James Evelyn Pilcher, Carlisle, Pa., the secretary of the association.

The Documents in the Case is the title of a somewhat graphic description of the case of Mrs. S—— and notices the marked and rapid improvement in her condition as evidenced by the differential blood counts. The Palisade Manufacturing Company, of Yonkers, may be applied to for information in regard to the treatment which proved so distinctly successful in this marked case of chloro-anemia. This unique legal looking folder is worth examining.

Fracture of the Olecranon is the subject of Chart No. 10, issued by Battle & Co., of St. Louis. It is clearly set forth and treatment in detail accompanies the drawing.

Polk's Medical Register of the United States and Canada is being revised for its eighth edition and will soon be put through the press. Every physician is interested in making this directory as accurate as possible and should respond promptly to requests from the publishers.

